# (*E*)-4-[(1-Benzyl-4-benzyl­idene-2,5-di­oxopyrrolidin-3-yl)meth­yl]benzalde­hyde 0.25-hydrate

**DOI:** 10.1107/S1600536812012160

**Published:** 2012-03-24

**Authors:** Tao Yu, Yimin Hu

**Affiliations:** a School of Chemistry and Materials Science, Anhui Normal University, Wuhu, Anhui 241000, People’s Republic of China

## Abstract

The crystal structure of the title compound, C_26_H_21_NO_3_·0.25H_2_O, reveals one stereogenic centre in the mol­ecule. Nevertheless, due to the observed centrosymmetric space group, both enanti­omers are present in the crystal packing. The water molecule of crystallisation is located on a crystallographic inversion center. The mol­ecule contains one five-membered ring (*A*) and three six-membered rings (benzyl ring *B*, benzyl­idene ring *C* and formyl­benzyl ring *D*). All four rings are not coplanar: the dihedral angles between rings *A* and *B*, *A* and *C*, and *A* and *D* are 70.35 (9), 33.8 (1) and 60.30 (9)°, respectively. In the crystal, pairs of weak C—H⋯O inter­actions lead to the formation of centrosymmetric dimers. Additional C—H⋯O inter­actions link the dimers into chains along [011].

## Related literature

For palladium-catalysed coupling reactions, see: Hu *et al.* (2011 ▶). For related active pharmaceutical compounds, see: Hu *et al.* (2009*a* ▶,*b* ▶). Ffor the physiological activity of dioxo­pyrrol­idinbenzaldehyde derivatives, see: Pitchumani & Vijaikumar (2010 ▶). F palladium-catalysed inter- and intra­molecular reactions, see: Hu *et al.* (2010*a* ▶,*b* ▶).
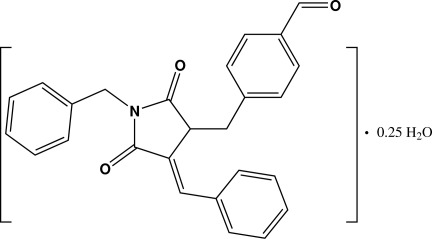


## Experimental

### 

#### Crystal data

C_26_H_21_NO_3_·0.25H_2_O
*M* *_r_* = 399.94
Triclinic, 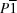

*a* = 7.7360 (15) Å
*b* = 12.441 (3) Å
*c* = 12.577 (3) Å
α = 64.14 (3)°
β = 81.14 (2)°
γ = 86.46 (3)°
*V* = 1076.2 (4) Å^3^
*Z* = 2
Mo *K*α radiation
μ = 0.08 mm^−1^
*T* = 291 K
0.28 × 0.22 × 0.20 mm

#### Data collection

Bruker SMART APEX CCD diffractometer
Absorption correction: multi-scan (*SADABS*; Bruker, 2000 ▶) *T*_min_ = 0.978, *T*_max_ = 0.984
8445 measured reflections
4224 independent reflections
3065 reflections with *I* > 2σ(*I*)
*R*_int_ = 0.027

#### Refinement

*R*[*F* ^2^ > 2σ(*F* ^2^)] = 0.061
*wR*(*F* ^2^) = 0.149
*S* = 1.09
4224 reflections
277 parameters
1 restraint
H-atom parameters constrained
Δρ_max_ = 0.21 e Å^−3^
Δρ_min_ = −0.20 e Å^−3^

### 

Data collection: *SMART* (Bruker, 2000 ▶); cell refinement: *SAINT* (Bruker, 2000 ▶); data reduction: *SAINT*; program(s) used to solve structure: *SHELXS97* (Sheldrick, 2008 ▶); program(s) used to refine structure: *SHELXL97* (Sheldrick, 2008 ▶); molecular graphics: *SHELXTL* (Sheldrick, 2008 ▶); software used to prepare material for publication: *SHELXTL*.

## Supplementary Material

Crystal structure: contains datablock(s) global, I. DOI: 10.1107/S1600536812012160/im2364sup1.cif

Structure factors: contains datablock(s) I. DOI: 10.1107/S1600536812012160/im2364Isup2.hkl

Supplementary material file. DOI: 10.1107/S1600536812012160/im2364Isup3.cml

Additional supplementary materials:  crystallographic information; 3D view; checkCIF report

## Figures and Tables

**Table 1:** Hydrogen-bond geometry (Å, °)

*D*—H⋯*A*	*D*—H	H⋯*A*	*D*⋯*A*	*D*—H⋯*A*
C10—H10*A*⋯O2^i^	0.98	2.56	3.419 (3)	147
C18—H18*A*⋯O2^i^	0.93	2.49	3.322 (3)	149
C19—H19*A*⋯O1^ii^	0.97	2.53	3.481 (3)	168

Symmetry codes: (i) 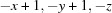; (ii) 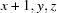.

**Fig. 1.**
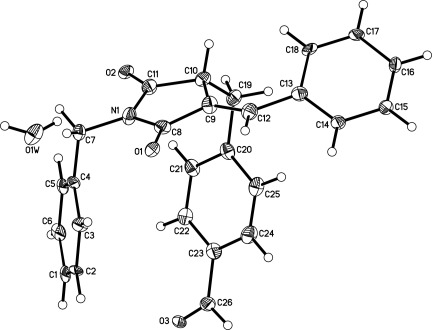
A view of the title compound showing the atom-numbering scheme and displacement ellipsoids drawn at the 30% probability level.

**Fig. 2.**
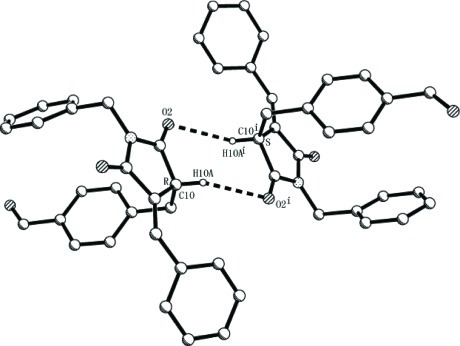
A view of a hydrogen bonded dimer of recemic molecules.

**Fig. 3.**
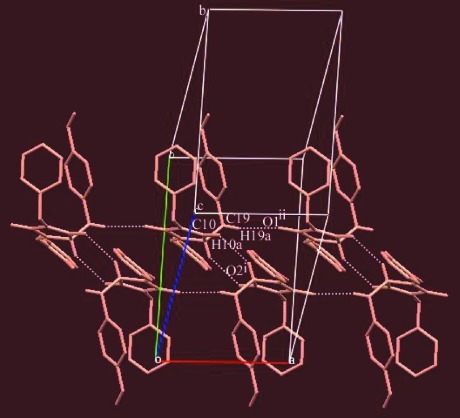
A view of the infinte one-dimensional chains of the dimeric substructures. [Symmetry codes: (i) 1-x, 1-y, -z; (ii) 1-x, y, z.]

## supplementary crystallographic information

### Comment

Palladium-catalyzed coupling reactions have become an important tool in modern organic synthetical chemistry (Hu *et al.*, 2011). A wide variety of active pharmaceutical compounds, natural substances and other complex organic molecules have been made economically accessible by this methodology (Hu *et al.*, 2009*a*, 2009*b*). Physiologically active dioxopyrrolidin-benzaldehyde derivatives are effective intermediates in the synthesis of many complex natural products (Pitchumani & Vijaikumar, 2010). We have reported some novel palladium-catalyzed inter- and intramolecular reactions of aryl halides with the olefins and enynes (Hu *et al.*, 2010*a*, 2010*b*). The reaction of *N*-acryloyl-*N*-benzylcinnamamide with 4-bromobenzaldehyde, in the presence of palladium(II) acetate and triphenylphosphine, in DMF at 373 K for 18 h, gave the unexpected title product.

The crystal structure data of molecule (I) reveals that all the bond lengths and angles have normal values. An asymmetric unit is composed of one title compound molecule and a quarter of a crystal water. The crystal water is located at the inversion center. The title compound molecule contains one five-membered (ring A (N1/C8/C9/C10/C11)) and three six-membered rings (ring B (C1/C2/C3/C4/C5/C6), ring C (C13/C14/C15/C16/C17/C18) and ring D (C20/C21/C22/C23/C24/C25)). All four rings are not coplanar. The dihedral angle between rings A and B, A and C, and A and D are 70.35 (9)°, 33.8 (1)°, and 60.30 (9)°, respectively (Fig 1). In the molecule there is one asymmetric carbon atom (C10). Nevertheless, due to the observed centrosymmetric space group both enantiomers are present in the crystal packing. In the crystal structure weak intermolecular C—H···O interactions (C10—H10*a*···O_2_^i ^(i: 1 - *x*,1 - *y*,-*z*)) lead to the formation of centrosymmetric dimers (Fig. 2). Additional C—H···O interactions (C19—H19*a*···O_1_^ii ^(ii: 1 + *x*, *y*,*z*)) produce one-dimensional infinite chains from these dimers (Fig. 3).

### Experimental

An oven-dried Schlenk tube was evacuated, filled with nitrogen, and then charged with *N*-acryloyl-*N*-benzylcinnamamide (2.91 g, 10 mmol), 4-bromobenzaldehyde (1.85 g, 10 mmol), tributylamine (3 ml), PPh_3_ (52.5 mg, 0.2 mmol), Pd(OAc)_2_ (24 mg, 0.1 mol), and DMF (10 ml) to give a yellow solution. The reaction mixture was heated to 373 K with stirring. After 18 h the reaction mixture was cooled to room temperature and the resulting yellow-orange mixture was diluted with Et_2_O (10 ml). The mixture was then washed with H_2_O (15 ml) and the aqueous layer was extracted with Et_2_O (20 ml). The combined organic layers were dried over MgSO_4_, filtered, and concentrated *in vacuo*. The crude material was purified by flash column chromatography on silica gel (petroleum ether: EtOAc = 9:1) and recrystalized from EtOAc (yield 3.16 g, 80%). Colorless crystals suitable for X-ray diffraction were obtained by recrystallization from a solution of the title compound in ethyl acetate over a period of one week.

### Refinement

H atoms were positioned geometrically and refined using a riding model with C—H = 0.93–0.97 Å, O—H = 0.85 Å, and with *U*_iso_(H) = 1.2 (1.5 for methyl groups) times *U*_eq_(C).

### Crystal data

**Table:**

C_26_H_21_NO_3_·0.25H_2_O	*Z* = 2
*M**_r_* = 399.94	*F*(000) = 421
Triclinic, *P*1	*D*_x_ = 1.234 Mg m^−^^3^
Hall symbol: -P 1	Mo *K*α radiation, λ = 0.71073 Å
*a* = 7.7360 (15) Å	Cell parameters from 3716 reflections
*b* = 12.441 (3) Å	θ = 2.1–24.7°
*c* = 12.577 (3) Å	µ = 0.08 mm^−^^1^
α = 64.14 (3)°	*T* = 291 K
β = 81.14 (2)°	Block, colourless
γ = 86.46 (3)°	0.28 × 0.22 × 0.20 mm
*V* = 1076.2 (4) Å^3^	

### Data collection

**Table:**

Bruker SMART APEX CCD diffractometer	4224 independent reflections
Radiation source: sealed tube	3065 reflections with *I* > 2σ(*I*)
Graphite monochromator	*R*_int_ = 0.027
phi and ω scans	θ_max_ = 26.0°, θ_min_ = 1.8°
Absorption correction: multi-scan (*SADABS*; Bruker, 2000)	*h* = −9→9
*T*_min_ = 0.978, *T*_max_ = 0.984	*k* = −13→15
8445 measured reflections	*l* = 0→15

### Refinement

**Table:**

Refinement on *F*^2^	Primary atom site location: structure-invariant direct methods
Least-squares matrix: full	Secondary atom site location: difference Fourier map
*R*[*F*^2^ > 2σ(*F*^2^)] = 0.061	Hydrogen site location: inferred from neighbouring sites
*wR*(*F*^2^) = 0.149	H-atom parameters constrained
*S* = 1.09	*w* = 1/[σ^2^(*F*_o_^2^) + (0.07*P*)^2^ + 0.3*P*] where *P* = (*F*_o_^2^ + 2*F*_c_^2^)/3
4224 reflections	(Δ/σ)_max_ < 0.001
277 parameters	Δρ_max_ = 0.21 e Å^−^^3^
1 restraint	Δρ_min_ = −0.20 e Å^−^^3^

### Special details

**Table:**

Geometry. All e.s.d.'s (except the e.s.d. in the dihedral angle between two l.s. planes) are estimated using the full covariance matrix. The cell e.s.d.'s are taken into account individually in the estimation of e.s.d.'s in distances, angles and torsion angles; correlations between e.s.d.'s in cell parameters are only used when they are defined by crystal symmetry. An approximate (isotropic) treatment of cell e.s.d.'s is used for estimating e.s.d.'s involving l.s. planes.Least-squares planes (*x*,*y*,*z* in crystal coordinates) and deviations from them (* indicates atom used to define plane)1.5177 (0.0088) *x* + 12.2930 (0.0068) *y* + 6.4535 (0.0131) *z* = 7.8343 (0.0042)* 0.0161 (0.0015) N1 * -0.0035 (0.0014) C8 * -0.0090 (0.0014) C9 * 0.0172 (0.0014) C10 * -0.0208 (0.0015) C11Rms deviation of fitted atoms = 0.01473.2207 (0.0064) *x* + 4.3390 (0.0100) *y* - 6.9725 (0.0098) *z* = 0.3855 (0.0095)Angle to previous plane (with approximate e.s.d.) = 70.35 (0.09)* 0.0022 (0.0016) C1 * -0.0017 (0.0015) C2 * 0.0004 (0.0014) C3 * 0.0003 (0.0014) C4 * 0.0002 (0.0014) C5 * -0.0015 (0.0015) C6Rms deviation of fitted atoms = 0.00131.5177 (0.0088) *x* + 12.2930 (0.0068) *y* + 6.4535 (0.0131) *z* = 7.8343 (0.0042)Angle to previous plane (with approximate e.s.d.) = 70.35 (0.09)* 0.0161 (0.0015) N1 * -0.0035 (0.0014) C8 * -0.0090 (0.0014) C9 * 0.0172 (0.0014) C10 * -0.0208 (0.0015) C11Rms deviation of fitted atoms = 0.01474.8630 (0.0064) *x* + 9.1574 (0.0092) *y* + 8.8027 (0.0100) *z* = 5.4849 (0.0080)Angle to previous plane (with approximate e.s.d.) = 33.76 (0.10)* 0.0019 (0.0017) C13 * -0.0066 (0.0017) C14 * 0.0011 (0.0016) C15 * 0.0093 (0.0016) C16 * -0.0141 (0.0016) C17 * 0.0086 (0.0017) C18Rms deviation of fitted atoms = 0.00821.5177 (0.0088) *x* + 12.2930 (0.0068) *y* + 6.4535 (0.0131) *z* = 7.8343 (0.0042)Angle to previous plane (with approximate e.s.d.) = 33.76 (0.10)* 0.0161 (0.0015) N1 * -0.0035 (0.0014) C8 * -0.0090 (0.0014) C9 * 0.0172 (0.0014) C10 * -0.0208 (0.0015) C11Rms deviation of fitted atoms = 0.01476.5252 (0.0054) *x* + 5.2018 (0.0111) *y* - 0.7915 (0.0119) *z* = 6.7075 (0.0096)Angle to previous plane (with approximate e.s.d.) = 60.30 (0.09)* -0.0090 (0.0016) C20 * 0.0056 (0.0016) C21 * -0.0033 (0.0016) C22 * 0.0043 (0.0018) C23 * -0.0082 (0.0018) C24 * 0.0105 (0.0016) C25Rms deviation of fitted atoms = 0.0073
Refinement. Refinement of *F*^2^ against ALL reflections. The weighted *R*-factor *wR* and goodness of fit *S* are based on *F*^2^, conventional *R*-factors *R* are based on *F*, with *F* set to zero for negative *F*^2^. The threshold expression of *F*^2^ > σ(*F*^2^) is used only for calculating *R*-factors(gt) *etc*. and is not relevant to the choice of reflections for refinement. *R*-factors based on *F*^2^ are statistically about twice as large as those based on *F*, and *R*- factors based on ALL data will be even larger.

### Fractional atomic coordinates and isotropic or equivalent isotropic displacement parameters (Å^2^)

**Table:**

	*x*	*y*	*z*	*U*_iso_*/*U*_eq_	Occ. (<1)
C1	−0.1529 (3)	0.6731 (2)	0.2926 (2)	0.0327 (5)	
H1A	−0.2385	0.6692	0.2499	0.039*	
C2	−0.1659 (3)	0.75660 (19)	0.33915 (19)	0.0304 (5)	
H2A	−0.2607	0.8082	0.3280	0.036*	
C3	−0.0374 (3)	0.76321 (19)	0.40232 (19)	0.0316 (5)	
H3A	−0.0460	0.8194	0.4331	0.038*	
C4	0.1036 (3)	0.6858 (2)	0.4193 (2)	0.0338 (5)	
H4A	0.1894	0.6901	0.4616	0.041*	
C5	0.1169 (3)	0.6020 (2)	0.3733 (2)	0.0331 (5)	
H5A	0.2116	0.5502	0.3847	0.040*	
C6	−0.0119 (3)	0.59562 (19)	0.3101 (2)	0.0310 (5)	
C7	0.0065 (3)	0.50546 (19)	0.25793 (19)	0.0283 (4)	
H7A	0.0740	0.4380	0.3057	0.034*	
H7B	−0.1087	0.4762	0.2616	0.034*	
C8	0.0049 (3)	0.6167 (2)	0.0375 (2)	0.0294 (5)	
C9	0.1367 (3)	0.6566 (2)	−0.0702 (2)	0.0323 (5)	
C10	0.3144 (3)	0.61565 (19)	−0.03003 (19)	0.0294 (5)	
H10A	0.3642	0.5601	−0.0627	0.035*	
C11	0.2696 (3)	0.5479 (2)	0.1037 (2)	0.0323 (5)	
C12	0.0939 (3)	0.7234 (2)	−0.1780 (2)	0.0386 (5)	
H12A	−0.0244	0.7411	−0.1801	0.046*	
C13	0.2048 (3)	0.7736 (2)	−0.2946 (2)	0.0360 (5)	
C14	0.1571 (3)	0.88196 (19)	−0.38198 (19)	0.0292 (5)	
H14A	0.0555	0.9198	−0.3665	0.035*	
C15	0.2610 (3)	0.9338 (2)	−0.49241 (19)	0.0305 (5)	
H15A	0.2292	1.0066	−0.5506	0.037*	
C16	0.4072 (3)	0.87893 (19)	−0.51516 (19)	0.0282 (5)	
H16A	0.4773	0.9147	−0.5887	0.034*	
C17	0.4552 (3)	0.76828 (19)	−0.42925 (19)	0.0312 (5)	
H17A	0.5542	0.7296	−0.4472	0.037*	
C18	0.3577 (3)	0.71743 (18)	−0.31989 (18)	0.0259 (4)	
H18A	0.3922	0.6455	−0.2619	0.031*	
C19	0.4489 (3)	0.71377 (19)	−0.0610 (2)	0.0308 (5)	
H19A	0.5544	0.6773	−0.0277	0.037*	
H19B	0.4791	0.7545	−0.1470	0.037*	
C20	0.3842 (3)	0.8034 (2)	−0.01497 (19)	0.0305 (5)	
C21	0.4122 (3)	0.7882 (2)	0.09694 (19)	0.0324 (5)	
H21A	0.4734	0.7216	0.1432	0.039*	
C22	0.3524 (3)	0.8682 (2)	0.14121 (19)	0.0294 (5)	
H22A	0.3711	0.8542	0.2175	0.035*	
C23	0.2649 (3)	0.9694 (2)	0.0748 (2)	0.0384 (5)	
C24	0.2369 (3)	0.9850 (2)	−0.0369 (2)	0.0380 (5)	
H24A	0.1751	1.0515	−0.0828	0.046*	
C25	0.2971 (3)	0.9064 (2)	−0.0814 (2)	0.0347 (5)	
H25A	0.2797	0.9217	−0.1582	0.042*	
C26	0.2021 (3)	1.0583 (2)	0.1198 (2)	0.0314 (5)	
H26A	0.1412	1.1233	0.0706	0.038*	
N1	0.0911 (2)	0.55614 (17)	0.13566 (17)	0.0330 (4)	
O1	−0.15018 (18)	0.62882 (12)	0.04401 (13)	0.0257 (3)	
O2	0.36924 (18)	0.49511 (13)	0.17642 (13)	0.0280 (3)	
O3	0.22208 (19)	1.05470 (13)	0.21319 (13)	0.0301 (4)	
O1W	0.5000	0.5000	0.5000	0.0428 (12)	0.50
H1X	0.4981	0.4401	0.5675	0.051*	0.25
H1Y	0.5958	0.5191	0.4527	0.051*	0.25

### Atomic displacement parameters (Å^2^)

**Table:**

	*U*^11^	*U*^22^	*U*^33^	*U*^12^	*U*^13^	*U*^23^
C1	0.0355 (12)	0.0359 (12)	0.0290 (11)	0.0174 (9)	−0.0162 (9)	−0.0146 (10)
C2	0.0302 (11)	0.0283 (11)	0.0261 (10)	0.0184 (9)	0.0041 (8)	−0.0107 (9)
C3	0.0320 (11)	0.0265 (11)	0.0258 (11)	−0.0154 (9)	0.0149 (9)	−0.0056 (9)
C4	0.0251 (11)	0.0360 (12)	0.0379 (12)	−0.0185 (9)	0.0152 (9)	−0.0176 (10)
C5	0.0315 (12)	0.0270 (11)	0.0308 (11)	−0.0142 (9)	0.0196 (9)	−0.0093 (9)
C6	0.0268 (11)	0.0245 (10)	0.0327 (11)	−0.0018 (8)	0.0168 (9)	−0.0108 (9)
C7	0.0262 (10)	0.0274 (10)	0.0261 (10)	−0.0055 (8)	0.0002 (8)	−0.0073 (9)
C8	0.0240 (11)	0.0340 (11)	0.0323 (11)	0.0104 (8)	−0.0104 (8)	−0.0156 (10)
C9	0.0280 (11)	0.0380 (12)	0.0297 (11)	0.0019 (9)	−0.0111 (9)	−0.0115 (10)
C10	0.0274 (11)	0.0283 (11)	0.0298 (11)	0.0118 (8)	−0.0133 (9)	−0.0086 (9)
C11	0.0296 (11)	0.0326 (11)	0.0293 (11)	0.0095 (9)	−0.0086 (9)	−0.0083 (9)
C12	0.0445 (14)	0.0367 (13)	0.0304 (12)	0.0091 (10)	−0.0099 (10)	−0.0103 (10)
C13	0.0341 (12)	0.0338 (12)	0.0347 (12)	0.0122 (10)	−0.0040 (9)	−0.0117 (10)
C14	0.0268 (11)	0.0302 (11)	0.0269 (11)	0.0112 (8)	−0.0063 (8)	−0.0096 (9)
C15	0.0331 (12)	0.0318 (11)	0.0217 (10)	0.0157 (9)	−0.0106 (9)	−0.0068 (9)
C16	0.0264 (10)	0.0260 (10)	0.0240 (10)	−0.0024 (8)	0.0000 (8)	−0.0040 (8)
C17	0.0322 (11)	0.0299 (11)	0.0286 (11)	0.0208 (9)	−0.0078 (9)	−0.0115 (9)
C18	0.0287 (10)	0.0203 (9)	0.0275 (11)	0.0146 (8)	−0.0162 (8)	−0.0070 (8)
C19	0.0325 (12)	0.0286 (11)	0.0287 (11)	0.0041 (9)	−0.0116 (9)	−0.0083 (9)
C20	0.0235 (10)	0.0394 (12)	0.0277 (11)	−0.0015 (9)	−0.0025 (8)	−0.0139 (10)
C21	0.0262 (11)	0.0437 (13)	0.0241 (11)	0.0082 (9)	−0.0086 (8)	−0.0113 (10)
C22	0.0263 (10)	0.0330 (11)	0.0286 (11)	−0.0021 (9)	−0.0093 (8)	−0.0110 (9)
C23	0.0428 (13)	0.0369 (13)	0.0304 (12)	0.0062 (10)	−0.0019 (10)	−0.0118 (10)
C24	0.0416 (13)	0.0356 (12)	0.0324 (12)	0.0098 (10)	−0.0174 (10)	−0.0081 (10)
C25	0.0294 (11)	0.0355 (12)	0.0326 (12)	−0.0022 (9)	−0.0094 (9)	−0.0068 (10)
C26	0.0240 (11)	0.0283 (11)	0.0368 (13)	0.0026 (8)	0.0029 (9)	−0.0119 (10)
N1	0.0298 (10)	0.0319 (10)	0.0277 (10)	0.0010 (8)	−0.0048 (7)	−0.0041 (8)
O1	0.0227 (7)	0.0253 (7)	0.0284 (8)	0.0028 (5)	−0.0112 (6)	−0.0088 (6)
O2	0.0210 (7)	0.0247 (7)	0.0334 (8)	0.0082 (6)	−0.0069 (6)	−0.0079 (6)
O3	0.0245 (7)	0.0294 (8)	0.0292 (8)	0.0100 (6)	0.0058 (6)	−0.0103 (6)
O1W	0.024 (2)	0.036 (3)	0.054 (3)	0.0020 (19)	−0.016 (2)	−0.003 (2)

### Geometric parameters (Å, º)

**Table:**

C1—C2	1.389 (3)	C13—C18	1.402 (3)
C1—C6	1.388 (3)	C14—C15	1.389 (3)
C1—H1A	0.9300	C14—H14A	0.9300
C2—C3	1.392 (3)	C15—C16	1.343 (3)
C2—H2A	0.9300	C15—H15A	0.9300
C3—C4	1.387 (3)	C16—C17	1.401 (3)
C3—H3A	0.9300	C16—H16A	0.9300
C4—C5	1.389 (3)	C17—C18	1.357 (3)
C4—H4A	0.9300	C17—H17A	0.9300
C5—C6	1.393 (4)	C18—H18A	0.9300
C5—H5A	0.9300	C19—C20	1.495 (3)
C6—C7	1.517 (3)	C19—H19A	0.9700
C7—N1	1.444 (3)	C19—H19B	0.9700
C7—H7A	0.9700	C20—C21	1.386 (3)
C7—H7B	0.9700	C20—C25	1.388 (3)
C8—O1	1.195 (3)	C21—C22	1.365 (3)
C8—N1	1.384 (3)	C21—H21A	0.9300
C8—C9	1.474 (3)	C22—C23	1.376 (3)
C9—C12	1.326 (3)	C22—H22A	0.9300
C9—C10	1.519 (3)	C23—C24	1.382 (3)
C10—C11	1.510 (3)	C23—C26	1.476 (3)
C10—C19	1.531 (3)	C24—C25	1.351 (4)
C10—H10A	0.9800	C24—H24A	0.9300
C11—O2	1.222 (3)	C25—H25A	0.9300
C11—N1	1.388 (3)	C26—O3	1.189 (3)
C12—C13	1.468 (3)	C26—H26A	0.9300
C12—H12A	0.9300	O1W—H1X	0.8500
C13—C14	1.390 (3)	O1W—H1Y	0.8500
			
C2—C1—C6	119.8 (2)	C15—C14—H14A	119.9
C2—C1—H1A	120.1	C13—C14—H14A	119.9
C6—C1—H1A	120.1	C16—C15—C14	120.0 (2)
C1—C2—C3	120.17 (19)	C16—C15—H15A	120.0
C1—C2—H2A	119.9	C14—C15—H15A	120.0
C3—C2—H2A	119.9	C15—C16—C17	120.7 (2)
C4—C3—C2	119.9 (2)	C15—C16—H16A	119.6
C4—C3—H3A	120.1	C17—C16—H16A	119.6
C2—C3—H3A	120.1	C18—C17—C16	120.16 (19)
C3—C4—C5	120.2 (2)	C18—C17—H17A	119.9
C3—C4—H4A	119.9	C16—C17—H17A	119.9
C5—C4—H4A	119.9	C17—C18—C13	119.79 (19)
C4—C5—C6	119.8 (2)	C17—C18—H18A	120.1
C4—C5—H5A	120.1	C13—C18—H18A	120.1
C6—C5—H5A	120.1	C20—C19—C10	112.94 (19)
C1—C6—C5	120.2 (2)	C20—C19—H19A	109.0
C1—C6—C7	120.6 (2)	C10—C19—H19A	109.0
C5—C6—C7	119.22 (19)	C20—C19—H19B	109.0
N1—C7—C6	112.17 (18)	C10—C19—H19B	109.0
N1—C7—H7A	109.2	H19A—C19—H19B	107.8
C6—C7—H7A	109.2	C21—C20—C25	116.5 (2)
N1—C7—H7B	109.2	C21—C20—C19	120.9 (2)
C6—C7—H7B	109.2	C25—C20—C19	122.6 (2)
H7A—C7—H7B	107.9	C22—C21—C20	121.8 (2)
O1—C8—N1	123.6 (2)	C22—C21—H21A	119.1
O1—C8—C9	128.5 (2)	C20—C21—H21A	119.1
N1—C8—C9	107.90 (18)	C21—C22—C23	121.0 (2)
C12—C9—C8	121.6 (2)	C21—C22—H22A	119.5
C12—C9—C10	130.6 (2)	C23—C22—H22A	119.5
C8—C9—C10	107.59 (18)	C22—C23—C24	117.3 (2)
C11—C10—C9	102.93 (18)	C22—C23—C26	122.0 (2)
C11—C10—C19	110.13 (18)	C24—C23—C26	120.7 (2)
C9—C10—C19	116.51 (18)	C25—C24—C23	121.8 (2)
C11—C10—H10A	109.0	C25—C24—H24A	119.1
C9—C10—H10A	109.0	C23—C24—H24A	119.1
C19—C10—H10A	109.0	C24—C25—C20	121.5 (2)
O2—C11—N1	123.0 (2)	C24—C25—H25A	119.2
O2—C11—C10	127.8 (2)	C20—C25—H25A	119.2
N1—C11—C10	109.08 (18)	O3—C26—C23	126.1 (2)
C9—C12—C13	129.9 (2)	O3—C26—H26A	117.0
C9—C12—H12A	115.0	C23—C26—H26A	117.0
C13—C12—H12A	115.0	C8—N1—C11	112.37 (18)
C14—C13—C18	119.1 (2)	C8—N1—C7	124.44 (19)
C14—C13—C12	118.4 (2)	C11—N1—C7	123.18 (19)
C18—C13—C12	122.4 (2)	H1X—O1W—H1Y	118.8
C15—C14—C13	120.1 (2)		
			
C6—C1—C2—C3	0.5 (3)	C15—C16—C17—C18	−2.6 (4)
C1—C2—C3—C4	−0.3 (3)	C16—C17—C18—C13	2.5 (4)
C2—C3—C4—C5	0.1 (3)	C14—C13—C18—C17	−1.0 (4)
C3—C4—C5—C6	−0.1 (3)	C12—C13—C18—C17	−179.8 (2)
C2—C1—C6—C5	−0.4 (3)	C11—C10—C19—C20	−60.7 (2)
C2—C1—C6—C7	−178.8 (2)	C9—C10—C19—C20	56.0 (3)
C4—C5—C6—C1	0.3 (3)	C10—C19—C20—C21	90.7 (3)
C4—C5—C6—C7	178.63 (18)	C10—C19—C20—C25	−90.6 (3)
C1—C6—C7—N1	84.9 (2)	C25—C20—C21—C22	2.0 (3)
C5—C6—C7—N1	−93.5 (2)	C19—C20—C21—C22	−179.2 (2)
O1—C8—C9—C12	6.1 (4)	C20—C21—C22—C23	−1.5 (4)
N1—C8—C9—C12	−176.0 (2)	C21—C22—C23—C24	1.4 (4)
O1—C8—C9—C10	−178.2 (2)	C21—C22—C23—C26	−178.8 (2)
N1—C8—C9—C10	−0.3 (2)	C22—C23—C24—C25	−1.9 (4)
C12—C9—C10—C11	177.4 (3)	C26—C23—C24—C25	178.3 (2)
C8—C9—C10—C11	2.2 (2)	C23—C24—C25—C20	2.5 (4)
C12—C9—C10—C19	56.8 (4)	C21—C20—C25—C24	−2.5 (3)
C8—C9—C10—C19	−118.4 (2)	C19—C20—C25—C24	178.7 (2)
C9—C10—C11—O2	178.1 (2)	C22—C23—C26—O3	1.7 (4)
C19—C10—C11—O2	−57.0 (3)	C24—C23—C26—O3	−178.5 (2)
C9—C10—C11—N1	−3.5 (2)	O1—C8—N1—C11	176.0 (2)
C19—C10—C11—N1	121.4 (2)	C9—C8—N1—C11	−2.0 (3)
C8—C9—C12—C13	178.6 (2)	O1—C8—N1—C7	−3.3 (4)
C10—C9—C12—C13	4.0 (5)	C9—C8—N1—C7	178.7 (2)
C9—C12—C13—C14	−148.5 (3)	O2—C11—N1—C8	−177.9 (2)
C9—C12—C13—C18	30.4 (4)	C10—C11—N1—C8	3.6 (3)
C18—C13—C14—C15	−0.5 (4)	O2—C11—N1—C7	1.4 (4)
C12—C13—C14—C15	178.4 (2)	C10—C11—N1—C7	−177.16 (19)
C13—C14—C15—C16	0.4 (4)	C6—C7—N1—C8	−86.5 (3)
C14—C15—C16—C17	1.2 (4)	C6—C7—N1—C11	94.3 (3)

### Hydrogen-bond geometry (Å, º)

**Table:**

*D*—H···*A*	*D*—H	H···*A*	*D*···*A*	*D*—H···*A*
C10—H10*A*···O2^i^	0.98	2.56	3.419 (3)	147
C18—H18*A*···O2^i^	0.93	2.49	3.322 (3)	149
C19—H19*A*···O1^ii^	0.97	2.53	3.481 (3)	168

Symmetry codes: (i) −*x*+1, −*y*+1, −*z*; (ii) *x*+1, *y*, *z*.
